# Evaluation of a new variant in the *aggrecan* gene potentially associated with chondrodysplastic dwarfism in Miniature horses

**DOI:** 10.1038/s41598-020-72192-3

**Published:** 2020-09-17

**Authors:** Danilo Giorgi Abranches de Andrade, Roberta Martins Basso, Angelo José Magro, Renée Laufer-Amorim, Alexandre Secorun Borges, José Paes de Oliveira-Filho

**Affiliations:** 1 School of Veterinary Medicine and Animal Science, São Paulo State University (Unesp), Botucatu, 18618-681 Brazil; 2 Institute for Biotechnology, São Paulo State University (Unesp), Botucatu, 18607-440 Brazil; 3 School of Agriculture, São Paulo State University (Unesp), Botucatu, 18610-034 Brazil

**Keywords:** Disease genetics, Gene amplification

## Abstract

Chondrodysplastic dwarfism in Miniature horses is an autosomal recessive disorder previously associated with four mutations (*D1*, *D2*, D3*, and *D4*) in the *aggrecan* (*ACAN*) gene. The aim of this study was to identify additional variants in the candidate *ACAN* gene associated with chondrodysplastic dwarfism in Miniature horses. Fifteen dwarf Miniature horses were found to possess only one of the dwarfism-causing variants, and two possessed none of the variants. The *ACAN* exons (EquCab3.0) of seven dwarf Miniature horses were sequenced. A missense SNP in coding exon 11 (g.95271115A > T, c.6465A > T—RefSeq XM_005602799.2), which resulted in the amino acid substitution p.Leu2155Phe (RefSeq XP_005602856.2), was initially associated with the dwarf phenotype. The variant was tested and found present in 14 dwarf foals as well as one parent of each, and both parents of a dwarf possessing two copies. Genetic testing of 347 phenotypically normal Miniature horses demonstrated that none had more than one of the dwarf alleles or c.6465A > T. However, a study of large breeds revealed the presence of c.6465A > T, which was present in homozygosis in two Mangalarga Marchador horses. We suggest that c.6465A > T as a marker of disequilibrium or complex interactions in the Miniature horse genome could contribute to the associated dwarfism.

## Introduction

Chondrodysplastic dwarfism is a genetic disorder that leads to a disproportionate reduction in body size and may negatively interfere with the development and reproduction of the affected individual^1^. In addition, aborted foetuses can be observed in some species as a clinical sign of this disease^2,3^. In horses, reports of dwarfism are mainly related to Friesian horses, Shetland ponies, and Miniature horses^2,4–6^. In all these breeds, the disorder presents an autosomal recessive inheritance pattern, but the causative mutations are in different genes. In Friesian horses, the *beta-1, 4 galactosyltransferase 7* (*B4GALT7*) gene is involved; in Miniature horses and Miniature Shetland ponies, the causative mutations are in the *aggrecan* (*ACAN*) gene^2,4,5^, or in the *short stature homeobox* (*SHOX*) gene^6^.

The *ACAN* gene encodes the large proteoglycan aggrecan, which provides a hydrated gel structure for the proper functioning of the articular cartilage, since the joints are dependent on the integrity of the extracellular matrix, which is composed of proteoglycans, hyaluronic acid, collagen type II, glycoproteins and elastic fibres. In addition, among the proteoglycans, aggrecan is the most crucial for the proper functioning of articular cartilage and is essential in chondroskeletal morphogenesis^7^.

Mutations in the *ACAN* gene also cause chondrodysplasia-like dwarfism in other species^3,8–10^. In humans, at least 25 pathological *ACAN* mutations have been identified as the cause of short stature^8^. Spondyloepiphyseal dysplasia type Kimberley, characterized by shortened limbs and trunk, is one of these diseases and is caused by a single base pair (bp) insertion in exon 12 of the *ACAN* gene^9^. In rats, cartilaginous matrix deficiency, characterized by cleft palate and short limbs, tail and muzzle, is caused by a seven bp deletion in the *ACAN* gene, resulting in a premature stop codon^10^. In turn, Dexter cattle foetuses affected by dwarfism related to alterations in the *ACAN* gene die around the seventh month of gestation^3^.

In Miniature horses, four *ACAN* gene variants (*D1*, *D2*, D3* and *D4*) have already been described as causative of chondrodysplastic dwarfism^2,5^. Most *D1* genotypes (*D1*/*D1*, *D1*/*D2*, *D1*/D3* and *D1*/*D4*) cause foetal death^2^, whereas the other genotypes (*D2*/*D2*, *D2*/D3*, *D2*/*D4*, D3*/D3*, D3*/*D4* and *D4*/*D4*) are involved in the birth of dwarf foals^2,5,11^. Clinical diagnosis of chondrodysplastic dwarfism in Miniature horses has already been described in Brazil^12^, and the *D4*/*D4* genotype was recently characterized in the same country^11^. However, it is still not possible to associate all dwarf Miniature horses with the previously described *ACAN* variants^2^. The aims of the present study were to locate and identify other variants in the candidate *ACAN* gene associated with chondrodysplastic dwarfism in Miniature horses and to verify the allele frequencies of the causative variants in a phenotypically normal population of Miniature horses in Brazil.

## Results

### *ACAN* variants detection

The genotyping for the four previously described causative variants (*D1*, *D2*, D3* and *D4*) of dwarfism^2^ in the 18 dwarf Miniature horses used in this study revealed that 13 animals were heterozygous for only *ACAN*-*D4* (N/*D4*), two animals were heterozygous for only *ACAN*-*D2* (N/*D2*), one animal had the *D2*/D3* genotype, and two animals did not possess any of the known dwarfism alleles (*D1*, *D2*, D3*, or *D4*). *ACAN* exons from seven dwarf Miniature horses (animals 1–7), which were admitted to the Large Animal Internal Medicine Section of São Paulo State University (Unesp), were sequenced except for two fragments (102 bp and 1,252 bp) in coding exon 11, which is located in a highly repetitive region. No alterations in the exon–intron junctions were found, and some genetic alterations were detected after comparison with the equine *ACAN* mRNA sequence (RefSeq XM_005602799.2—EquCab3.0). A total of six synonymous SNPs and four missense SNPs were found in the *ACAN* gene of dwarf Miniature horses (Table 1).

**Table 1:** Synonymous and missense SNPs found in the *ACAN* mRNA sequencing of seven affected Miniature horses.

Animal(s)	Genotype	Coding exon	mRNA (XM_005602799.2)	Protein (XP_005602856.2)	SNP type
1; 3	A/A	11	c.2406G > A	p.Thr802Thr	Synonymous
4; 5; 6; 7	G/A	11	c.2406G > A	p.Thr802Thr	Synonymous
4	T/A	11	c.3453 T > A	p.Ser1151Ser	Synonymous
2	A/A	11	c.4950C > A	p.Leu1650Leu	Synonymous
1; 3; 4; 5	C/A	11	c.4950C > A	p.Leu1650Leu	Synonymous
6	C/T	11	c.5145C > T	p.Pro1715Pro	Synonymous
1; 3; 4; 5; 6; 7	C/T	11	c.5883C > T	p.Ser1961Ser	Synonymous
1	T/T	15	c.7836C > T	p.Thr2612Thr	Synonymous
3; 4; 5; 6; 7	C/T	15	c.7836C > T	p.Thr2612Thr	Synonymous
1; 2; 3; 4; 5; 6; 7	C/C	11	c.3277A > C	p.Ile1093Leu	Missense
1	T/T	11	c.6128C > T	p.Ala2043Val	Missense
3; 4; 5; 6; 7	C/T	11	c.6128C > T	p.Ala2043Val	Missense
2	T/T	11	c.6465A > T	p.Leu2155Phe	Missense
1; 3; 4; 5; 6; 7	A/T	11	c.6465A > T	p.Leu2155Phe	Missense
1; 2; 3; 4; 5; 6; 7	G/G	11	c.7096A > G	p.Arg2366Gly	Missense

**Figure 1:**
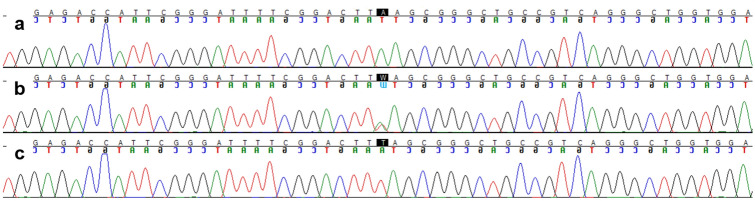
Partial electropherograms of the *ACAN*:c.6465A > T (RefSeq XM_005602799.2—EquCab3.0) region obtained after Sanger sequencing. (**a**) Normal horse. (**b**) Carrier (heterozygous). (**c**) Affected foal.

To check the relationship of these missense SNPs with the dwarf phenotype, the parents of the affected Miniature horses were genotyped according to these SNPs and the variants *D1*, *D2*, D3*, and *D4*. The SNPs c.3277A > C and c.7096A > G (RefSeq XM_005602799.2—EquCab3.0) were also found to be homozygosis in 30.8% and 100% of the parents, respectively. Since the parents were phenotypically normal and these two SNPs were found to be homozygosis, c.3277A > C and c.7096A > G were discarded as responsible for dwarfism in the Miniature horses with inconclusive genotypes in the present study. SNP c.6128C > T (RefSeq XM_005602799.2—EquCab3.0) was not found to be homozygosis in any of the parents of the affected animals; however, 87.5% of the parents were heterozygous for both this SNP and the *D4* mutation. Since these parents were phenotypically normal, c.6128C > T was also discarded. Conversely, all the parents were homozygous for the reference allele (c.6465A—RefSeq XM_005602799.2—EquCab3.0) or heterozygous for the SNP c.6465A > T. The parents that were heterozygous for the c.6465A > T variant did not possess any of the *ACAN* causative variants (*D1*, *D2*, D3*, and *D4*). In addition, six of seven dwarf offspring of the c.6465A > T heterozygous parents were also heterozygous for c.6465A > T and for the *ACAN*-*D4* causative variant previously described^2^, and the other dwarf foal was homozygous for SNP c.6465A > T. Therefore, the analysis of the sequencing results between the affected animals and their parents revealed that c.6465A > T (g.95271115A > T; p.Leu2155Phe—RefSeq XM_005602799.2; RefSeq XP_005602856.2—EquCab3.0) would be a novel variant potentially associated with chondrodysplastic dwarfism in Miniature horses (Fig. 1, Supplementary Figure S1).

The two dwarf Miniature horses assessed in the present study, which did not possess the *D1*, *D2*, D3*, and *D4* variants (animals 2 and 15), were found to be homozygous for c.6465A > T (RefSeq XM_005602799.2—EquCab3.0). In addition, the 13 animals that were heterozygous for only *ACAN*-*D4* (N/*D4*) and the two animals that were heterozygous for only *ACAN*-*D2* (N/*D2*) were also heterozygous for c.6465A > T (Table 2).

**Table 2:** General information about the 18 dwarf Miniature horses, including age, gender, farm, and genetic test results, for *D1*, *D2*, D3*, *D4*, and c.6465A > T.

Animal	Age	Gender	Farm	*D1*	*D2*	D3*	*D4*	c.6465A > T
1	One day	Male	A	N/N	N/N	N/N	N/*D4*	A/T
2	One day	Male	A	N/N	N/N	N/N	N/N	T/T
3	One day	Female	A	N/N	N/N	N/N	N/*D4*	A/T
4	One day	Male	A	N/N	N/N	N/N	N/*D4*	A/T
5	One day	Female	B	N/N	N/N	N/N	N/*D4*	A/T
6	One day	Male	A	N/N	N/N	N/N	N/*D4*	A/T
7	One day	Male	A	N/N	N/N	N/N	N/*D4*	A/T
8	One day	Female	A	N/N	N/N	N/N	N/*D4*	A/T
9	One day	Male	A	N/N	N/N	N/N	N/*D4*	A/T
10	One day	Female	A	N/N	N/N	N/N	N/*D4*	A/T
11	One day	Female	A	N/N	N/N	N/N	N/*D4*	A/T
12	One day	Female	A	N/N	N/N	N/N	N/*D4*	A/T
13	One day	Male	A	N/N	N/N	N/N	N/*D4*	A/T
14	One day	Female	A	N/N	N/N	N/N	N/*D4*	A/T
15	One year	Male	C	N/N	N/N	N/N	N/N	T/T
16	One year	Male	D	N/N	N/*D2*	N/D3*	N/N	A/A
17	Adult	Female	E	N/N	N/*D2*	N/N	N/N	A/T
18	Adult	Female	E	N/N	N/*D2*	N/N	N/N	A/T

All the missense SNPs were analysed by PolyPhen-2, SIFT and PROVEAN. The amino acid residue substitution p.Leu2155Phe (RefSeq XP_005602856.2; c.6465A > T, RefSeq XM_005602799.2—EquCab3.0) was predicted to be “probably damaging” (0.994), and it was noted that this substitution “affects protein function” (0.00) according to PolyPhen-2 and SIFT, respectively. A PROVEAN analysis suggested that p.Leu2155Phe was “neutral” using the default cutoff (score -2.222, cutoff = -2.5). However, the prediction for p.Leu2155Phe would be “deleterious” if the cutoff were higher (cutoff = -1.3), similar to the sensitivity of the detection. In addition, the aggrecan amino acid sequence from an affected Miniature horse homozygous for c.6465A > T (RefSeq XM_005602799.2—EquCab3.0) was aligned with those of 21 other species. Comparison of the amino acid sequences in this section for 21 species (Supplementary Figure S2) showed a conservation of leucine at this position in 13 but substitution with threonine or glutamine in 8 others.

Finally, 93 horses from different large breeds (23 Mangalarga Marchador, 23 Warmblood, 23 Thoroughbred and 24 Quarter horses) were tested for SNP c.6465A > T (RefSeq XM_005602799.2—EquCab3.0). Two Mangalarga Marchador horses were homozygous, and heterozygous animals were found in all tested breeds (Table 3). We also sequenced 26 horses from the same breeds for *D1*, *D2*, D3*, and *D4* (9 Mangalarga Marchador, 5 Warmblood, 6 Thoroughbred, and 6 Quarter horses). All of them were N/N except one Mangalarga Marchador horse that was N/*D2* (Table 4).

**Table 3:** Frequencies of the genotypes at position c.6465 (RefSeq XM_005602799.2) after sequencing results from 93 horses of four different large breeds.

Breed	A/A	A/T	T/T
Mangalarga Marchador	30.4% (7/23)	60.8% (14/23)	8.8% (2/23)
Warmblood	91.3% (21/23)	8.7% (2/23)	0.0% (0/23)
Thoroughbred	73.9% (17/23)	26.1% (6/23)	0.0% (0/23)
Quarter horse	79.2% (19/24)	20.8% (5/24)	0.0% (0/23)

**Table 4:** Frequencies of animals with *D1*, *D2*, D3*, and *D4* variants after sequencing results from 26 horses of four different large breeds.

Variant	Genotype	Mangalarga Marchador (n = 9) (%)	Warmblood (n = 5) (%)	Thoroughbred (n = 6) (%)	Quarter horse (n = 6) (%)
*D1*	N/N	0.0	0.0	0.0	0.0
	N/*D1*	0.0	0.0	0.0	0.0
	*D1*/*D1*	0.0	0.0	0.0	0.0
*D2*	N/N	0.0	0.0	0.0	0.0
	N/*D2*	11.1 (1/9)	0.0	0.0	0.0
	*D2*/*D2*	0.0	0.0	0.0	0.0
D3*	N/N	0.0	0.0	0.0	0.0
	N/D3*	0.0	0.0	0.0	0.0
	D3*/D3*	0.0	0.0	0.0	0.0
*D4*	N/N	0.0	0.0	0.0	0.0
	N/*D4*	0.0	0.0	0.0	0.0
	*D4*/*D4*	0.0	0.0	0.0	0.0

### Dwarf Miniature horses

Eighteen Miniature horses with dwarfism were used in this study, belonging to five different farms (Table 2), and were tested for existing *ACAN* alleles^2^ (Table 1). Fifteen dwarf Miniature horses (animals 1–15) were admitted at the Large Animal Internal Medicine Section of São Paulo State University (Unesp). Of these, 14 animals were one day old, of which two died during medical care and 12 were euthanized on the second day of life due to future dwarfism complications. One horse (animal 15; homozygous for *ACAN*:c.6465A > T) was one year old and was discharged after improvement in respiratory clinical signs; we have no knowledge of his history after hospital discharge. During sample collection for the *ACAN* variants prevalence estimation, three Miniature horses were also phenotypically identified as dwarfs (animals 16–18).

**Figure 2:**
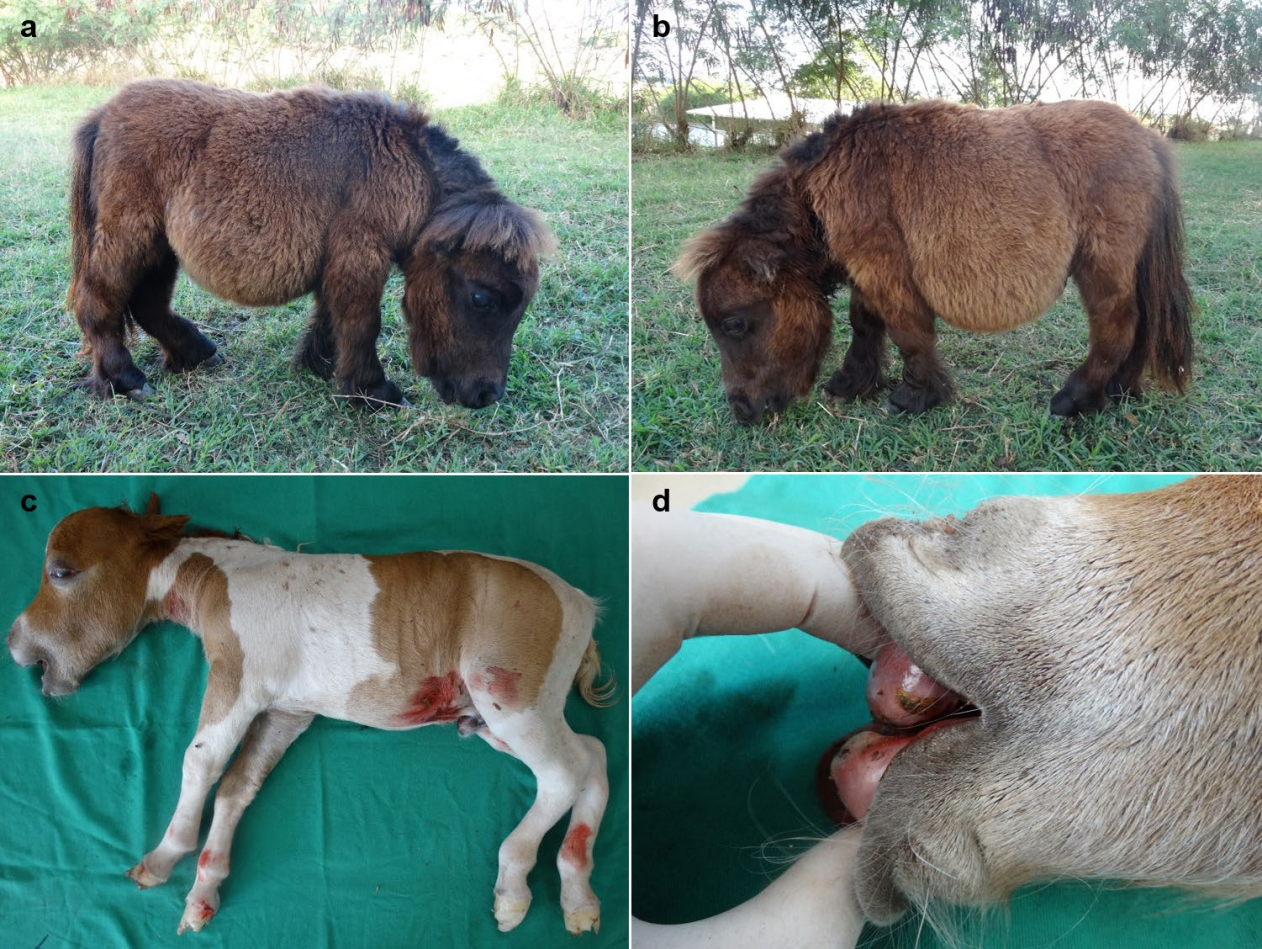
Clinical signs of dwarfism in Miniature horses. (**a**), (**b**) and (**c**) Domed head; enlarged eye sockets with prominent eyes; shortened neck; shortened limbs relative to overall body size; and bowed limbs, mainly the hind limbs. (**d**) Mandibular prognathism. (**a**) and (**b**) represent the same Miniature horse (animal 15) as in (**c**) and (**d**) (animal 2). These two Miniature horses did not possess *D1*, *D2*, D3*, and *D4* variants and were homozygous for *ACAN*:c.6465A > T.

Physical examination of animals 1–16 revealed a disproportionate and short body, shortened limbs relative to overall body size, a disproportionately large cranium, bilateral enlarged eye sockets with prominent eyes, a shortened nasal bone, mandibular prognathism, a shortened neck, and bowed limbs, mainly the hind limbs (Fig. 2). The two affected mares that were evaluated at a farm both were heterozygous for *ACAN*-*D2* and for *ACAN*:c.6465A > T (animals 17 and 18). It was determined that their phenotypic characteristics were different from each other, despite having the same genotype, and they were mildly affected compared with the other affected animals in this study. One had a disproportionate and short body, shortened limbs relative to overall body size and a shortened neck, while the other had shortened limbs relative to overall body size and bilateral enlarged eye sockets with prominent eyes. As their phenotypic characteristics were only mildly observable, their owner did not notice that they were dwarfs, so they had both been used for breeding.

In the radiographic examinations of the 15 affected Miniature horses that were referred to the veterinary hospital (animals 1–15), irregularities in the limbs and in the head (e.g., subchondral bone irregularities in the long bones and mandibular prognathism) were observed. In addition, tracheal collapse (Supplementary Figure S3) was radiographically observed in the one-year-old Miniature horse with dwarfism (animal 15; homozygous for *ACAN*:c.6465A > T). This animal presented with respiratory distress, tachypnoea, inspiratory honking noises and dysphagia. The other dwarfs had no clinical signs of tracheal collapse, but airway endoscopy revealed mild tracheal collapse in eight of ten dwarf Miniature horses (animals 5–14). Moreover, other abnormalities were observed during airway endoscopy examination, such as dorsal displacement of the soft palate (5/10) and thickening of both arytenoid cartilages (4/10). Histological evaluation of the proximal portion of the metatarsus revealed lesions consistent with chondrodysplasia. Disorganization of the cartilage structure and a decrease in the extracellular matrix were observed in all of the affected Miniature horses (Supplementary Figure S4) that were examined (n = 14).

### Population screening

Of the 347 phenotypically normal Miniature horses assigned to determine the allele frequencies of the *ACAN* causative variants, 34.6% (120/347) were males and 65.4% (227/347) were females. These animals belonged to nine farms; all but one had a history of births of affected animals (Table 5).

**Table 5:** Percentages of *D1*, *D2*, D3*, *D4*, or c.6465A > T heterozygous Miniature horses by farm (n = 347).

Farm	Sampling	Heterozygous (%)	Dwarf births in the past
A	79	78.5	Yes
B	59	91.5	Yes
D	17	64.7	Yes
E	82	78.0	Yes
F	3	100.0	Yes
G	30	83.3	Yes
H	18	38.9	No
I	43	86.0	Yes
J	16	56.3	Yes

Among the phenotypically normal Miniature horses, none was identified with more than one of the *D1*, *D2*, D3*, *D4* or c.6465A > T variants. The cumulative frequency of the four causative alleles of dwarfism (*D1*, *D2*, D3*, and *D4*) was 0.280 (q), which suggests a heterozygous (2pq) rate of 40.3% and a predicted rate of dwarf Miniature horses (q^2^) of 7.8%. The frequencies of the four *ACAN* gene variants associated with dwarfism are shown in Table 6. The allele frequency of c.6465A > T (RefSeq XM_005602799.2—EquCab3.0) was 0.112, and if this variant could be associated with dwarfism, the cumulative frequency would be 0.392 (q), which could suggest a heterozygous (2pq) rate of 47.7% and a predicted rate of dwarf Miniature horses (q^2^) of 15.4%.

**Table 6:** Allele frequencies of *D1*, *D2*, D3*, and *D4* variants in the *ACAN* gene in Brazil (n = 347) and in the USA (n = 361).

Brazil (n = 347)		USA (n = 361)^2^	
Variant	Frequency	Variant	Frequency
Normal (N)	0.720	Normal (N)	0.84
g.95291270del (*D1*)	0.003	g.95291270del (*D1*)	0.03
g.95284530C > T (*D2*)	0.017	g.95284530C > T (*D2*)	0.09
g.95282140C > G (D3*)	0.016	g.95282140C > G (D3*)	0.02
g.95257458_95257500del (*D4*)	0.244	g.95257458_95257500del (*D4*)	0.03

## Discussion

The initial genotyping results of the 18 dwarf Miniature horses assessed in this study, similar to the findings of a previous study^2^, reinforced the suspicion of the existence of additional mutations responsible for dwarfism in Miniature horses. Although other genes have already been associated with dwarfism in horses^4,6^, we decided to investigate novel variants in the candidate *ACAN* gene, since mutations in this gene are related to the birth of dwarf Miniature horses and a dwarf Miniature Shetland pony^2,5^. In addition, several genetic skeletal diseases in humans have been identified as a result of *ACAN* mutations leading to a broad phenotypic spectrum, such as spondyloepiphyseal dysplasia type Kimberley^9^, spondyloepimetaphyseal dysplasia^13^, and familial osteochondritis dissecans^14^.

As in a previous study^2^, we were not able to sequence the initial region of coding exon 11 (RefSeq XM_005602799.2—EquCab3.0), and with our methodology, it was not possible to cover two fragments (102 bp and 1,252 bp) with an interval of 318 bp. However, a comparison between the mRNA sequencing results of seven dwarf Miniature horses and the equine *ACAN* mRNA sequence (RefSeq XM_005602799.2—EquCab3.0) revealed four missense SNPs, which were compared with the DNA sequence of the affected Miniature horse parents. Three SNPs were discarded due to genetic conditions, and the SNP c.6465A > T (g.95271115A > T; p.Leu2155Phe—RefSeq XM_005602799.2; RefSeq XP_005602856.2—EquCab3.0) was primarily considered a novel variant potentially associated with chondrodysplastic dwarfism in Miniature horses, although a causative variant could reside in the unsequenced region of coding exon 11, and c.6465A > T could simply be a marker of linkage disequilibrium with other functionally important and presumably causative variant.

*ACAN*-*D1* consists of a single nucleotide deletion in coding exon 2 (g.95291270del), which results in a stop codon and a truncated protein (p.Lys82X)^2^. On the other hand, *ACAN*-*D2* and *ACAN*-D3* are characterized by missense SNP substitutions, the first in coding exon 6 (g.95284530C > T), which causes an amino acid change (p.Val424Met) in aggrecan globular domain 1 (G1)^2^, and the second in coding exon 7 (g.95282140C > G), which results in an amino acid change (p.Ala505Pro) in the interglobular domain (IGD) of aggrecan^2,5^. This last SNP was also described in a Miniature Shetland pony with dwarfism in Germany^5^. Previously, this variant was designated *ACAN*-*D3* and consisted of a deletion in coding exon 11. However, this result was found to be a technical artefact, and the novel D3* designation was assigned to differentiate the correct causative variant^2^. Finally, *ACAN*-*D4* consists of a 21-bp deletion in coding exon 14 (g.95257458_95257500del) that affects aggrecan globular domain 3 (G3) (p.Phe2017-Asp2023del)^2^.

Aggrecan is a chondroitin sulfate proteoglycan of the lectican family composed of different domains^7^, and many causes of dwarfism in humans and animals are associated with mutations in this extracellular matrix protein^13–16^. However, in contrast to the aggrecan alterations already described in Miniature horses^2^, the p.Leu2155Phe (*ACAN*:c.6465A > T—RefSeq XP_005602856.2—EquCab3.0) substitution is located in the chondroitin sulphate (CS) domain. This domain is the largest aggrecan domain and may harbour chains of approximately 100 CS molecules^7^. These CS molecules are negatively charged and are essential to the most important function of aggrecan as a structural proteoglycan, which is the capacity to retain a large amount of water in the extracellular matrix. Notably, dwarfism in Miniature Zebu calves can be caused by a single base pair insertion in the CS domain, which leads to a truncated protein^17^.

Although the p.Leu2155Phe (*ACAN*:c.6465A > T—RefSeq XP_005602856.2—EquCab3.0) substitution does not produce a truncated aggrecan, an analysis based on computational tools^18–20^ indicated the influence of this substitution on proteoglycan function, which corroborated the genetic findings. The PROVEAN score for p.Leu2155Phe (-2.222) was higher than the default cutoff (-2.5), which could indicate that this amino acid substitution would not interfere with protein function. However, a similar PROVEAN finding was observed for *D2* protein function, which was primarily estimated to be neutral based on the physical and chemical properties of the amino acid change (score -0.874)^2^. Unfortunately, a nonhomologous region to the Miniature horse aggrecan CS domain was found in the structural databases. Thus, it was impossible to model the aggrecan region containing the amino acid substitution p.Leu2155Phe and, consequently, analyse the effects of *ACAN*:c.6465A > T (RefSeq XM_005602799.2—EquCab3.0) on the structure/function of the CS domain. However, the substitution of an aliphatic apolar amino acid residue (Leu) by an aromatic amino acid residue (Phe) has suggestive potential of inducing significant structural/functional modifications in the Miniature horse aggrecan CS domain.

Despite the presence of the *ACAN*:c.6465A > T (RefSeq XM_005602799.2—EquCab3.0) variant in heterozygosity in horses belonging to the other four tested large breeds and in homozygosity in two Mangalarga Marchador horses, we suggest that further studies are needed to analyse the potential association of this variant with dwarfism in Miniature horses and the role of *D1*, *D2*, D3*, and *D4* in large-breed horses. Seemingly, Miniature horses are phylogenetically more closely related to Mangalarga Marchador horses compared with the other large breeds (Warmblood, Thoroughbred, and Quarter horses)^21^, which could explain the higher frequencies of *ACAN* gene variants in Mangalarga Marchador horses. Since *ACAN*-*D2* was found in heterozygosity in one Mangalarga Marchador horse, despite the small number of sampled animals, we suggest that the potential association between *ACAN*:c.6465A > T (RefSeq XM_005602799.2—EquCab3.0) and dwarfism in Miniature horses would continue to be relevant; moreover, *ACAN*:c.6465A > T has provided population, genetic, and informatics prediction evidence that it could be a potential causative variant of dwarfism in this breed^22^. Indeed, a mutation may not always cause the same effects, or it may lead to different phenotypes of the same disorder in different individuals^23,24^. Several genetic modifiers can produce unexpected phenotypes of the primary disease-causing variant^25^, and the genetic background of Miniature horses and Shetland ponies, which were selected for diminutive size over the years, differs from horses of large breeds^26–29^ and may generate a predisposition to the condition characterized as dwarfism due to *ACAN* variants.

In addition, *D1*, *D2*, D3*, and *D4* can affect height measurements at the withers in Miniature horses; therefore, *ACAN* variant carriers are smaller than non-carriers^30^. In those other large breeds, *ACAN* variants might be involved in height at the withers or with the aetiology of arthropathies^9,14^. As there have been no reports on dwarfism in the tested large breeds (Mangalarga Marchador, Warmblood, Thoroughbred, and Quarter horses) and we do not know the height at the withers of these horses and their history of articular diseases, more studies are needed to clarify the genetic pathway of aggrecan in Miniature horses and in horses of other breeds.

The clinical signs observed in the Miniature horses with dwarfism described here were similar to those previously reported^2,5,11,12^. As *ACAN*-*D1* was not observed in the dwarf Miniature horses of the present study, the clinical signs that this variant can cause (cleft palate with protruding tongue, large abdominal hernia and embryonic or late term loss)^2^ were not reported in the affected animals of the present study. Therefore, our results also suggest that the genotype of an animal cannot be assumed only from the phenotypic characteristics, except for genotypes associated with *ACAN*-*D1*^2^.

Mutations in genes that encode proteins associated with CS synthesis and sulfation can result in different severities of skeletal dysplasias^31^. This characteristic can explain the phenotypic differences between the two mares that were heterozygous for *ACAN*-*D2* and *ACAN*:c.6465A > T, since *ACAN*:c.6465A > T is a substitution located in the CS domain. In addition, the clinical signs of *ACAN*-*D2* are less deleterious than those related to *D1*, D3* and *D4*^2^, and PROVEAN analysis indicated that *D2*^2^ and *ACAN*:c.6465A > T were “neutral” using the default cutoff, which may suggest mild phenotypic characteristics.

The radiographic findings of the 15 affected Miniature horses that were referred to the veterinary hospital (animals 1–15) were similar to those of a previous study^11^, and they were not consistent with skeletal atavism (e.g., a complete fibula and ulna were not found)^6,32^. The ten animals that were subjected to airway endoscopy did not show inspiratory honking noises at the auscultation of the midcervical tracheal region; however, the airway findings suggested that in young adulthood, they could demonstrate clinical signs of tracheal collapse on auscultation (e.g., mild tracheal collapse in eight of ten dwarf Miniature horses). Chondrodysplasia was reported in all 14 dwarf Miniature horses that had histological evaluation performed. Although a study carried out in Germany^5^ reported no apparent histopathological findings of chondrodysplasia in a Miniature Shetland pony with the D3*/D3* genotype, *ACAN*-D3* was classified as causative of chondrodysplasia-like dwarfism in Miniature horses^2^.

The data shown in Table 6 suggest that the birth rate of affected animals in Brazil may be mainly due to the *ACAN*-*D4* variant, since this variant had the highest frequency (0.244) in the studied population. In the USA, a study using 361 randomly selected horses submitted for testing by private Miniature horse owners found that *ACAN*-*D2* was the most prevalent variant (0.09), followed by *ACAN*-*D1* (0.03), *ACAN*-*D4* (0.03) and *ACAN*-D3* (0.02). The cumulative frequency of these dwarfism alleles was 0.163, and the predicted rate of affected horses (q^2^) was 2.7%^2^. The Brazilian predicted rate of affected horses (q^2^) would be 7.8% considering only *D1*, *D2*, D3*, and *D4* variants, or 15.4% including the *ACAN*:c.6465A > T findings. Therefore, in both scenarios, the predicted rate of affected Miniature horses in Brazil might be more influenced by the other breeds used in the origin and formation of Brazilian Miniature horses than by American Miniature horses, which were also part of the origin of Brazilian Miniature horses and are imported as an option for expanding the gene pool of the Brazilian animals. In addition, the variant prevalence differences might be explained because different foundation horses were used in Miniature horses within the two countries, and the Brazilian Miniature horse population might be more inbred than the American Miniature horse population.

The previously described causative variants of dwarfism (*D1*, *D2*, D3* and *D4*) were identified in the studied population of Miniature horses. In addition, another potentially causative variant of this disorder was found (c.6465A > T, g.95271115A > T; p.Leu2155Phe—RefSeq XM_005602799.2; RefSeq XP_005602856.2—EquCab3.0). This substitution is a novel finding, since it affects the aggrecan CS domain, a protein region never previously associated with dwarf births in Miniature horses. The phenotypic characteristics of heterozygous dwarfs for *ACAN*-*D4* and for *ACAN*:c.6465A > T and homozygous dwarfs for *ACAN*:c.6465A > T were similar to those of the affected animals with *D2*/*D2*, *D2*/D3*, *D2*/*D4*, D3*/*D4*, and *D4*/*D4* genotypes. The cumulative allele frequency of the five variants was 0.392 in Miniature horses in Brazil, and *ACAN*-*D4* and *ACAN*:c.6465A > T were more prevalent. Based on our findings, we suggest that control measures to identify heterozygous animals based on genetic testing should be adopted to minimize the significant economic losses and casualties related to affected foals.

## Methods

The animal study was approved by the Ethics Committee for the Use of Animals in Research (CEUA) of the School of Veterinary Medicine and Animal Science of São Paulo State University—UNESP on February 9th, 2017 (nº 219/2016-CEUA). All methods were carried out in accordance with the guidelines and regulations of CEUA, and samples were collected under a strict confidentiality agreement to ensure the anonymity of establishments, owners, and animals. In addition, all owners allowed the use of their animals in this study through an informed consent statement.

### *ACAN* variant detection

Miniature horse individuals with clinical signs of dwarfism (n = 18) were used in this study. These dwarfs were numbered in a sequence from 1 to 18 on the basis of their physical examination date. All of the affected Miniature horses (n = 18) were subjected to genetic testing for *D1*, *D2*, D3* and *D4* according to methodology previously described^11^. The parents of 14 Miniature horses with dwarfism were also subjected to genetic testing for *D1*, *D2*, D3* and *D4* with the same methodology^11^. Materials from the parents of four affected animals were not available.

Seventeen out of 18 dwarfs did not present the combined genotypes of previously described mutations (*D1*, *D2*, D3* or *D4*) associated with this disorder. We then chose the first seven dwarfs admitted to the Large Animal Internal Medicine Section of São Paulo State University (Unesp) for *ACAN* mRNA sequencing. The other nine affected Miniature horses did not have their *ACAN* mRNA sequenced, because genetic testing for the newly found and potentially associated with dwarfism variant *ACAN*:c.6465A > T (RefSeq XM_005602799.2—EquCab3.0) was already standardized, and their genotypes were consistent with the dwarf phenotype after the results of the *D1*, *D2*, D3*, *D4* and c.6465A > T genetic tests.

Total RNA was isolated from the skin samples collected at necropsy of seven affected Miniature horses. The RNeasy Fibrous Tissue Mini Kit (QIAGEN) was used for total RNA isolation following the manufacturer’s instructions, except for the cell lysis step, which was performed on a Precellys instrument (Bertin Instruments). The relative purity and quality of the isolated RNA was determined by a NanoDrop 2000 Spectrophotometer (Thermo Scientific), and the ratio of A260–A280 nm exceeded 1.8 for all preparations. To ensure the complete removal of traces of genomic DNA, 1 µg of total RNA was incubated with RQ1 RNase-Free DNase (Promega). First-strand cDNA synthesis was performed with 500 ng of total RNA per 45 µl of reaction using random hexamers and the ImProm-II Reverse Transcription System (Promega), following the manufacturer’s instructions. The cDNA samples were stored at -20 °C.

The primer pairs designed to sequence the 16 coding exons of the predicted *ACAN* mRNA (RefSeq XM_005602799.2—EquCab3.0) were previously described^11^; these primers were designed for evaluation of the exons and the exon–intron junctions of the *ACAN* gene. The PCR was standardized to a total volume of 25 μL containing 2 μL of cDNA, 0.2 μM of each primer, 12.5 μL of GoTaq Green Master Mix (Promega), and 9.5 μL of nuclease-free water. In addition, a no-template control reaction was performed to check for the possible presence of contamination in the PCR preparations. The amplification conditions were as follows: initial denaturation at 95 °C for 5 min; followed by 40 cycles of denaturation at 95 °C for 30 s, annealing at 63 °C for 60 s, and extension at 72 °C for 60 s; and a final extension at 72 °C for 5 min. Amplicons were analysed by 1.5% agarose gel electrophoresis, purified, and subjected to Sanger sequencing.

To sequence the *ACAN* mRNA, 10 μL of purified PCR product and 5 μL of forward or reverse primers and BigDye Terminator Cycle Sequencing Kit were used (Thermo Scientific). The sequences were determined using a genetic analyser (3,500 Series Genetic Analyzer, Thermo Scientific). The obtained sequences and electropherograms were analysed using Geneious and Sequencher 5.1 (Gene Codes Corporation), and they were compared to the equine *ACAN* mRNA sequence (RefSeq XM_005602799.2—EquCab3.0). The comparisons of the sequenced products allowed the interpretation of the results.

### Validation of the variant potentially associated with dwarfism (*ACAN*:c.6465A > T)

#### Variant analysis

The missense SNPs found in the *ACAN* mRNA sequences of the dwarf Miniature horses were compared with the corresponding *ACAN* sequences of the parents of the dwarf Miniature horses. For this, the blood DNA of the parents of the affected Miniature horses was amplified as previously described^11^, according to the positions of the missense SNPs. Then, the PCR products were purified, and Sanger sequencing was performed according to the aforementioned methodology. In addition, this analysis was assigned to estimate the mode of disease inheritance potentially associated with *ACAN*:c.6465A > T.

#### Protein study

All amino acid substitutions caused by missense SNPs found in the affected Miniature horses were analysed with PolyPhen-2^18^, SIFT^19^ and PROVEAN^20^ to assess the impact of the amino acid substitutions on the biological function and structure of the *ACAN* protein. In addition, the aggrecan amino acid sequence from a dwarf Miniature horse (homozygous for *ACAN*:c.6465A > T) was aligned with those of 21 other species.

#### Comparison with other breeds

Ninety-three horses from different large breeds (23 Mangalarga Marchador, 23 Warmblood, 23 Thoroughbred, and 24 Quarter horses) were tested for *ACAN*:c.6465A > T (RefSeq XM_005602799.2—EquCab3.0) according to the aforementioned methodology. In addition, 26 horses from these breeds (9 Mangalarga Marchador, 5 Warmblood, 6 Thoroughbred, and 6 Quarter horses) were tested for *D1*, *D2*, D3*, and *D4* as previously described^11^. All the samples were randomly chosen and belonged to the DNA stock of our laboratory. We did not obtain body measurements or articular disease histories of these horses.

### Dwarf Miniature horses

Physical examinations were performed on all dwarf Miniature horses (n = 18). Radiographs of the limbs (tibia/fibula and radius/ulna) and head were performed on the affected Miniature horses that were referred to the veterinary hospital (n = 15). Ten neonatal dwarfs underwent airway endoscopy. Of the 14 animals that were one day old, two died during medical care, and 12 were euthanized on the second day of life due to future dwarfism complications; all of them underwent necropsy, in which the proximal portion of the metatarsus was collected for histopathology, and skin samples were collected for RNA isolation. Tissue samples of the metatarsus were formalin fixed and decalcified in HNO_3_ prior to paraffin embedding. For histopathological examination, the fixed tissues were routinely processed, stained with haematoxylin and eosin, and examined by light microscopy.

### Population screening

Genetic testing (*D1*, *D2*, D3* and *D4*) according to the methodology previously described^11^ and using the DG_ACAN_Eq_F13 and DG_ACAN_Eq_R13 primer pair^11^ for *ACAN*:c.6465A > T (RefSeq XM_005602799.2—EquCab3.0) was performed in 347 phenotypically normal Miniature horses randomly sampled from nine farms in Brazil. The parents of the 14 neonatal Miniature horses with dwarfism admitted to the Large Animal Internal Medicine Section of São Paulo State University (Unesp) were included in this sampling. Eight farms were in São Paulo state, and one farm was in Rio Grande do Sul state. The screening was assigned to calculate the prevalence of the *ACAN* variants (*D1*, *D2*, D3*, *D4* and c.6465A > T) in Brazil.

## Supplementary information

Supplementary file1
