# Supplementary material for: Evaluation of a new variant in the aggrecan gene potentially associated with chondrodysplastic dwarfism in Miniature horses
Source: Sci Rep. 2020 Sep 17;10:15238. doi: 10.1038/s41598-020-72192-3 (PMC7499210; doi:10.1038/s41598-020-72192-3)
Supplement: Supplementary file 1 — Supplementary file1 [file 41598_2020_72192_MOESM1_ESM.pdf]

Supplementary information

Evaluation of a new variant in the *aggrecan* gene potentially associated with chondrodysplastic dwarfism in Miniature horses

Danilo Giorgi Abranches de Andrade[1], Roberta Martins Basso[1], Angelo José Magro[2,3], Renée Laufer-Amorim[1], Alexandre Secorun Borges[1], José Paes de Oliveira-Filho[1*]

[1]São Paulo State University (Unesp), School of Veterinary Medicine and Animal Science, Botucatu, 18618-681, Brazil.

[2]São Paulo State University (Unesp), Institute for Biotechnology, Botucatu, 18607-440, Brazil.

[3]São Paulo State University (Unesp), School of Agriculture, Botucatu, 18610-034, Brazil.

*jose.oliveira-filho@unesp.br

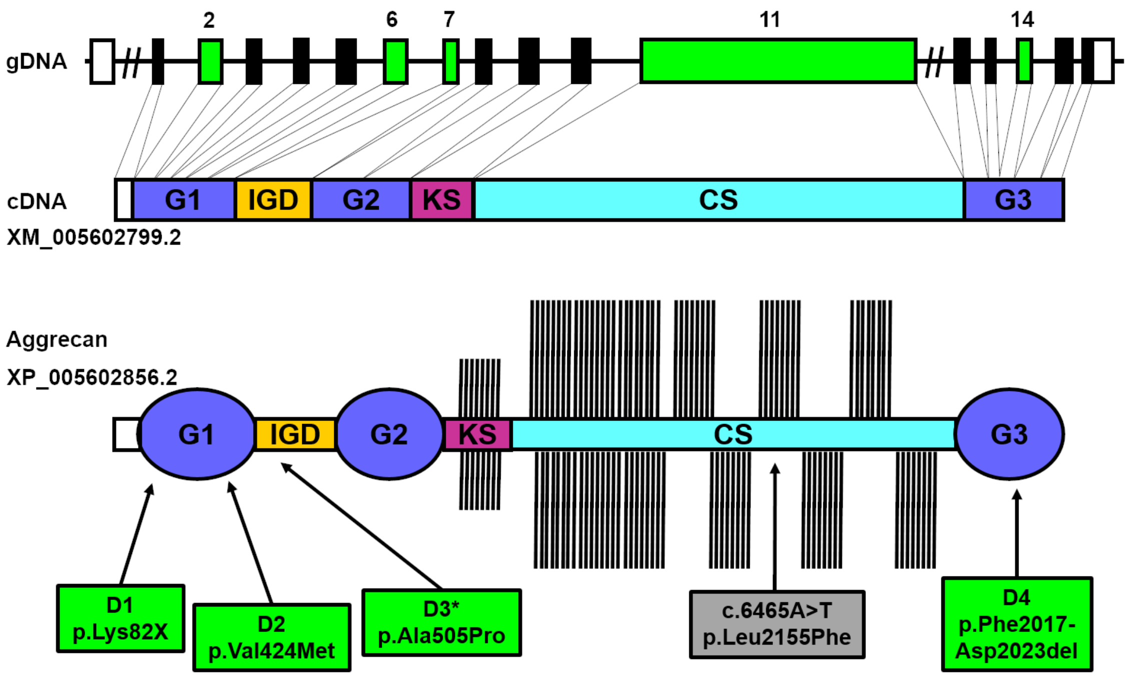

Supplementary Figure S1. Schematic structure of aggrecan and the location of the variants *D1*, *D2*, *D3**, *D4* and c.6465A>T. Genomic DNA (gDNA): the white rectangles indicate the untranslated regions; green rectangles indicate the coding exons where *D1*, *D2*, *D3**, *D4* and c.6465A>T variants are located (2, 6, 7, 11 and 14); black rectangles indicate the coding exons without known variants associated with dwarfism. Coding DNA (cDNA – RefSeq XM_005602799.2) and aggrecan (RefSeq XP_005602856.2): G1, globular domain 1; IGD, interglobular domain; G2, globular domain 2; KS, keratan sulfate domain; CS, chondroitin sulfate domain; G3, globular domain 3. Green boxes with arrows represent the *D1*, *D2*, *D3** and *D4* locations throughout the amino acid chain. Gray box with arrow represents the c.6465A>T (p.Leu2155Phe) location. *D1*, *D2*, *D3** and *D4* first described in Eberth *et al*. (2018). Images generated by Microsoft PowerPoint 2013 and GIMP 2.10.2.

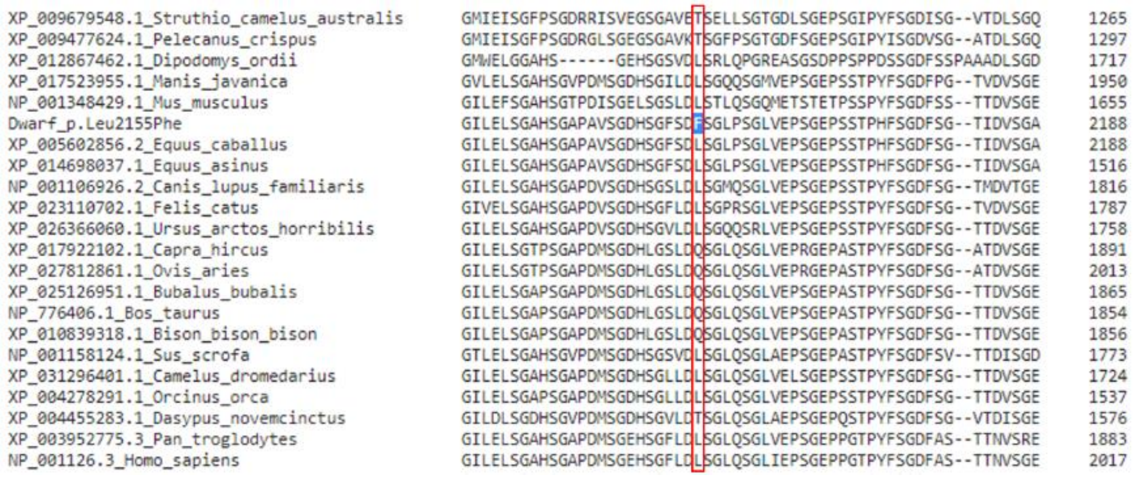

Supplementary Figure S2. Alignment of the aggrecan sequences of 19 mammalian species, two avian species and an affected Miniature horse homozygous for the SNP c.6465A>T between positions 2138–2195 (RefSeq XP_005602856.2). The red box indicates position 2155, and highlighted in blue is p.Leu2155Phe in the Miniature horse homozygous for the SNP c.6465A>T. This alignment was performed with the Clustal Omega tool (https://www.ebi.ac.uk/Tools/msa/clustalo/).

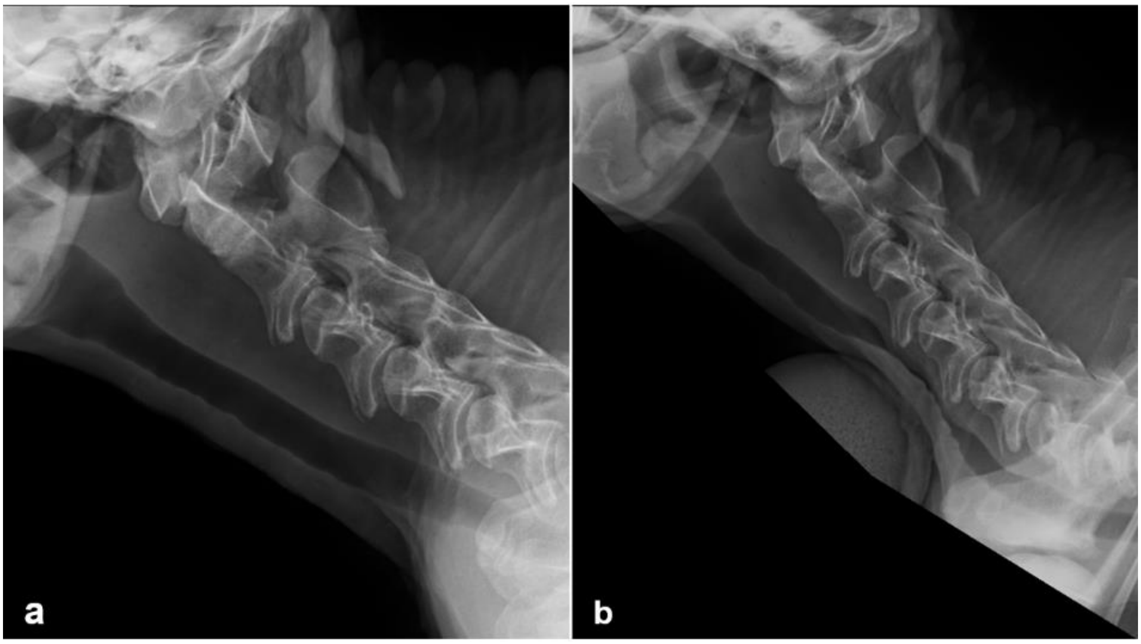

Supplementary Figure S3. Radiographic findings in a Miniature horse with dwarfism and homozygous for the SNP c.6465A>T. (**a**) Lateral radiograph before compression of

the cervical trachea portion (animal 15). (**b**) Lateral radiograph showing tracheal collapse at the cervical trachea portion (animal 15).

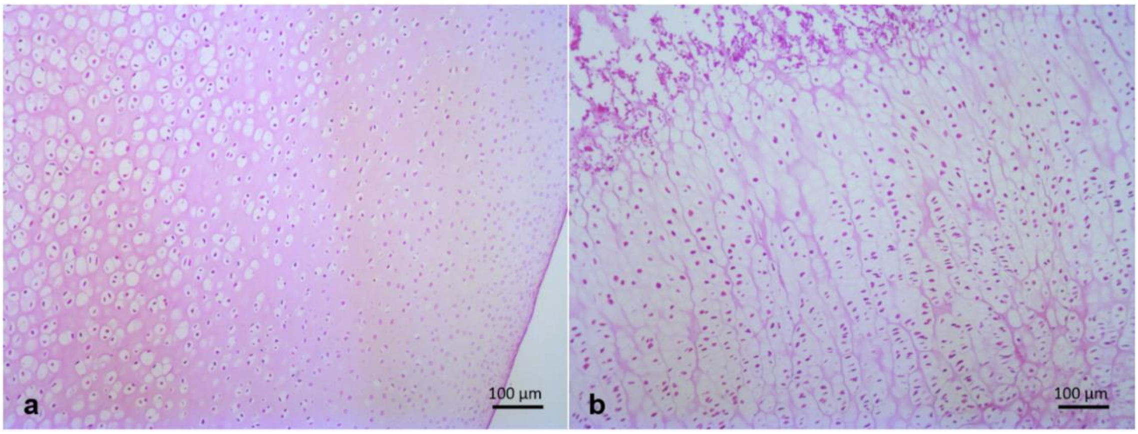

Supplementary Figure S4. Histopathological findings using H&E staining in a Miniature horse with dwarfism and homozygous for the SNP c.6465A>T. Histopathological images of the articular cartilage (**a**) and of the physis cartilage (**b**) of a two-day-old Miniature horse with dwarfism (animal 2). Note the chondrocyte disorganization and the decreased cartilage matrix.
